# Research Progress on the Role and Mechanism of Action of Activin A in Brain Injury

**DOI:** 10.3389/fnins.2018.00697

**Published:** 2018-10-09

**Authors:** Xiaojuan Su, Lingyi Huang, Dongqiong Xiao, Yi Qu, Dezhi Mu

**Affiliations:** ^1^Department of Pediatrics, West China Second University Hospital, Sichuan University, Chengdu, China; ^2^Key Laboratory of Birth Defects and Related Diseases of Women and Children, Sichuan University, Ministry of Education, Chengdu, China; ^3^Department of Stomatology, West China College of Stomatology, Sichuan University, Chengdu, China

**Keywords:** activin A, transforming growth factor β, activin A/Smad, brain injury, neuroprotection, target therapy

## Abstract

Activin A belongs to the transforming growth factor superfamily and has a variety of biological functions. Studies have revealed that activin A can regulate the body's immune and inflammatory responses and participate in the regulation of cell death. In addition, activin A also has neurotrophic function and plays an important role in the repair of brain damage. This article summarizes recent advances in understanding the role and mechanism of action of activin A in brain injury and provides new hints into the application of activin A in the treatment of brain injury.

## Introduction

Brain injury is a typical functional disorder of the nervous system, with many types of pathogenic factors involved and a complex pathogenesis. The main pathogenesis involves massive cell death in injured brain areas and surrounding tissue, which leads to tissue damage and eventually destruction, mainly caused by systematic inflammation, including ischemia and traumatic brain injury (Chandra et al., 2018).

Activin A, a member of the transforming growth factor beta (TGF-β) superfamily, regulates the body's immune and inflammatory responses, and participates in the regulation of cell death. In addition, activin A has a neurotrophic function and plays an important role in repair following brain damage (Ageta and Tsuchida, 2011). For example, a transgenic mouse study showed that activin A exerts a critical role in neuroprotection following various types of brain damage, by regulating spine formation and adult neurogenesis (Müller et al., 2006).

In recent years, the role of activin A and its molecular mechanisms in brain injury have been studied extensively. Activin A may thus represent a promising therapeutic target, and knowledge about its function may provide some hints for clinical treatment and drug discovery. The present review discusses recent progress in the study of the role and mechanism of action of activin A in brain injury.

## Structural characteristics and regulation of activin A activity

### Structural features of activin A

activin A is a widely expressed homodimer that is composed of two β A chains. Sequence analysis showed that the β subunit has the typical structural features of the TGF-β superfamily, i.e., the C-terminal active portion of the molecule has nine conserved cysteine residues (Wang X. et al., 2016). In addition, the 14-kDa mature human β A chain of activin A has 100% amino acid sequence identity in cattle, cats, mice, pigs, etc., indicating its highly conserved structure (Tanimoto et al., 1991).

### Regulation of activin A activity

**Figure 1:**
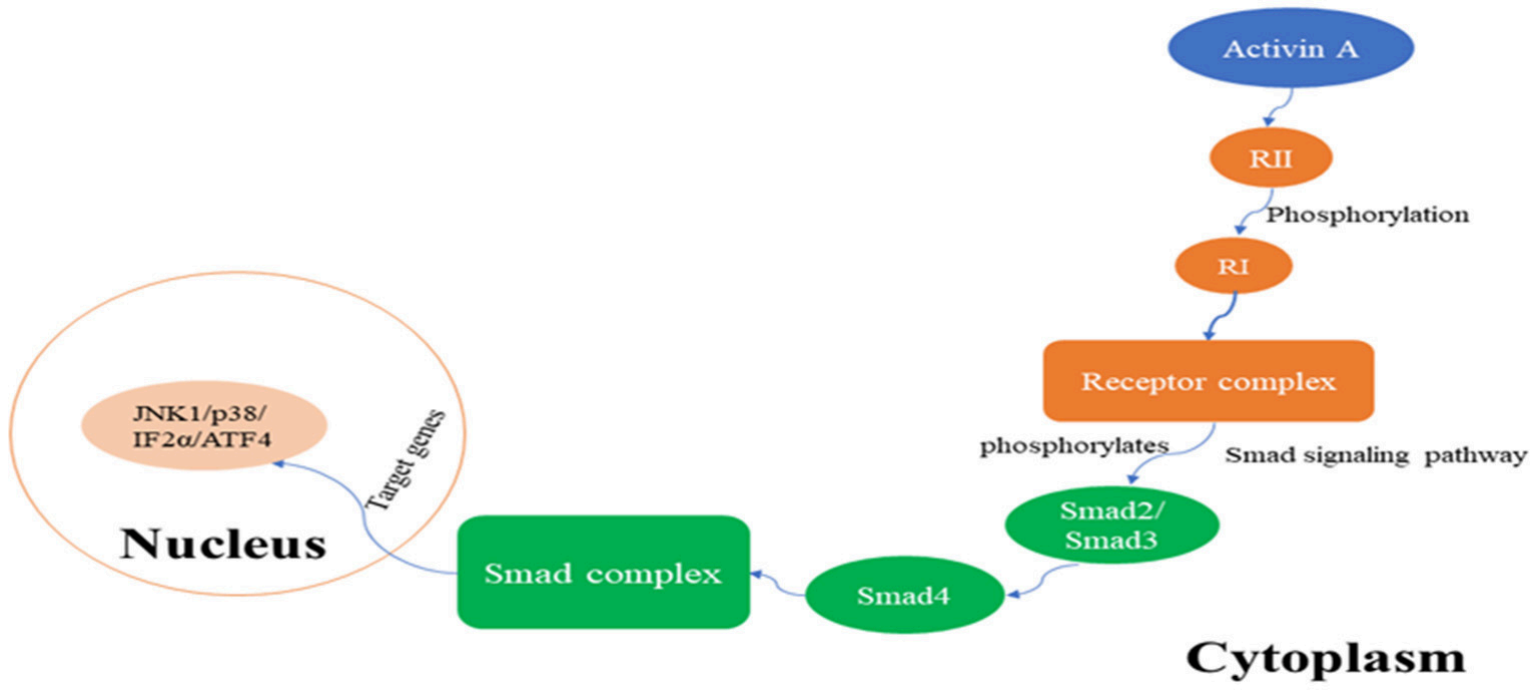
The activin A/Smad signaling pathway.

The receptor for activin A is a serine threonine protein kinase, and there are three types of receptors: type I (RI), type II (RII), and type III (RIII). RI and RII are mainly involved in the regulation of activin A activity, whereas RIII is not indispensable for its activity. RI is also known as activin receptor-like kinase 5 (ALK5) and can be phosphorylated by RII (de Kroon et al., 2015). Upon signal transduction, activin A first binds to RII, which in turn activates RI to form the receptor complex. After being activated, signaling of the formed receptor complex results in activation of the Smad signaling pathway, which includes phosphorylation of transcription factors Smad2 or Smad3 by ALK5 protein kinase, and leads to the transcriptional regulation of activin A response genes. Furthermore, phosphorylated Smad2 or Smad3 bind to Smad4, and the resulting complex translocates from the cytoplasm to the nucleus, where the Smad complex interacts in a cell-specific manner with various other transcription factors, thus exerting its biological activity (Peterson et al., 2012; Figure 1). In addition to the Smad-dependent signaling pathways, there are activin A pathways that are Smad-independent and mainly include the nuclear factor-κB pathway, extracellular signal-regulated kinase (ERK1/2) pathway, ubiquitin-proteolytic pathways, mitogen-activated protein kinase (MAPK) pathways, and other signal transduction pathways (Derynck and Zhang, 2003; Kim et al., 2015). The activin A/Smad molecular pathway is shown in Figure 1.

Studies have found that activin A activity is regulated by numerous factors. For example, it is negatively regulated by some factors, including the bone morphogenetic protein (BMP), BMP and activin membrane-bound inhibitor, glycan, and uterine natural killer, which limit its ability to induce the assembly of receptor complexes (Shi et al., 2009; Upton et al., 2009; Mai et al., 2014; Peng et al., 2015). Other factors, like 2-macroglobulin, follistatin, and follistatin-related genes, limit activin A bioavailability by binding to it (Asashima et al., 1991; Mather, 1996; de Kretser et al., 2012). In contrast, it has been reported that, in the early stages of tissue damage, TGF-β, epidermal growth factor, and platelet-derived growth factor are released from platelets, thus triggering the upregulation of activin A and leading to protection of the damaged nerve (Hübner et al., 1999; Wang and Ge, 2004; Protic et al., 2017). These factors were also found to promote the expression of the activin β A subunit in both cultured fibroblasts and keratinocytes (Huang et al., 2004). *In vitro* studies have shown that high levels of interleukin (IL)-1 and tumor necrosis factor alpha (TNF-α) are expressed in polymorphonuclear leucocytes and macrophages, both of which can also promote activin A secretion (Arai et al., 2011; Kelly et al., 2016). Moreover, recent studies have discovered a new group of intracellular proteins, termed activin A receptor-interacting proteins, which interact with activin A RII and regulate an activin A-dependent intracellular signaling process, influenced by activin A histological distribution and biological activity (Liu et al., 2009; Liu H. Y. et al., 2013; Qi et al., 2013; Table 1). The molecules that regulate activin A activity are summarized in Table 1.

**Table 1:** Molecules regulating activin A activity.

	**Promoting factors**	**References**	**Inhibitory factors**	**References**
Regulatory molecules for activin A activity	TGF-β	Hübner et al., 1999	BMP	Shi et al., 2009
	EGF	Protic et al., 2017	BAMBI	Mai et al., 2014
	PDGF	Wang and Ge, 2004	glycan	Peng et al., 2015
	IL-1	Arai et al., 2011	UNK	Upton et al., 2009
	TNF-α	Kelly et al., 2016	2-macroglobulin	Asashima et al., 1991
	ARIP1/ARIP2	Liu et al., 2009; Liu H. Y. et al., 2013; Qi et al., 2013	Follistatin/follistatin-related genes	Mather, 1996; de Kretser et al., 2012

*ARIP1/ARIP2, activin receptor-interacting protein 1/2; BAMBI, BMP and activin membrane-bound inhibitor; BMP, bone morphogenetic protein; EGF, epidermal growth factor; IL-1, interleukin 1; TGF-β, transforming growth factor beta; TNF-α, tumor necrosis factor alpha; PDGF, platelet-derived growth factor; UNK, uterine natural killer*.

## Activin A and brain injury

activin A and its receptors are widely expressed in brain tissue. Studies have shown that the expression of activin A is upregulated after nerve cells are subjected to acute injury from various sources (Mukerji et al., 2007). The neuroprotective effect of activin A after brain injury occurs mainly through its anti-inflammatory activity; however, the inhibition of the secretion of certain reactive proteins, reduction of cytotoxic brain edema, anti-oxidation, inhibition of free radical aggregation, upregulation of brain-derived neurotrophic factor and induction of its synthesis, as well as the antagonization of excitatory amino acid-induced neurotoxicity, among other functions, render activin A an important molecule with an endogenous protective role (Wang Q. et al., 2016). Moreover, a study found that activin A increases the number of synapses and the length of the dendritic aponeurosis neck in hippocampal neurons cultured *in vitro*, while it also increases the activity of neuronal voltage-gated Na+/K+ channels and triggers the maturation of synapses (Manickam and Tulsawani, 2014).

### Activin A and hypoxic-ischemic brain injury

The rat model of cerebral ischemic and hypoxic injury induces the overexpression of follistatin, activin A, and BMP-4, depending on development and age, all of which are protective against nerve injury. As the rat grows older, the expression of follistatin and activin A decreases gradually, whereas BMP-4 expression decreases significantly in adulthood. (Tian et al., 2014). During ischemic and hypoxic injury, the exogenous use of activin A was shown to inhibit the expression of caspase-3 and apoptosis in neural cells (He et al., 2011). Besides, activin A also negatively regulate autophagy, which was found to inhibit the c-Jun N-terminal kinase 1 (JNK) and p38 MAPK signaling pathways during cerebral ischemia (Xue et al., 2017). In addition, a study found that activin A is upregulated in the early stage of acute ischemic brain injury, whereby it exerts its neuroprotective role by downregulating nitric oxide levels and increasing superoxide dismutase activity and neuronal tolerance to ischemic injury, through the activin A/Smad signaling pathway (Nakajima et al., 2014). In a mouse model of ischemia/reperfusion, it was found that intracerebroventricular injection of activin A inhibits neuronal apoptosis and significantly reduces the infarct size (Ma et al., 2016).

In a PC12-cell oxygen-glucose deprivation (OGD) and endoplasmic reticulum stress (ERS) lesion model, the protein levels of activin A and phosphorylated Smad3 (p-Smad3) were significantly upregulated (Guo et al., 2014). Treatment with exogenous activin A was shown to further increase activin A and p-Smad3 protein levels, as well as cell viability, and to significantly reduce the number of apoptotic nuclei and the levels of the C/EBP homologous protein and caspase-12. These findings indicate that activin A/Smad signaling exerts neuroprotective effects by inhibiting ERS-mediated apoptosis during OGD. The study also found that the expression of microtubule-associated protein light chain 3 (LC3) and of Beclin1 is significantly upregulated after OGD in PC12 cells and increases with the extension of OGD duration. Interestingly, application of exogenous activin A significantly inhibits LC3II and Beclin1 protein levels (Wang et al., 2016a). Together, the *in vivo* and *in vitro* study suggest that activin A exerts its neuroprotection role mainly through negatively regulate apoptotic and autophagic pathway.

### Activin A/Smad pathway and focal cerebral ischemia in rats

When transient cerebral ischemia and hypoxia occurs, the expression of activin A, as a neuronal survival factor, as well as that of its effectors RII or Smad3, is significantly upregulated. It was found that activin A and Smad3 are mainly expressed in the cytoplasm and nucleus, whereas RII is mainly expressed in the cytoplasm and membrane of the cells. This change in expression levels occurs specifically in neurons, suggesting that the activin A/Smad pathway is activated after focal cerebral ischemia (Mukerji et al., 2009).

It was also reported that activin A, as a neuronal autocrine factor, may act on the neuron itself and mediate signal transduction through the activin A/Smad pathway after ischemia (Hiratochi et al., 2007). In addition, in PC12 OGD models, blockade of activin A RII site in the activin A transmembrane signal transduction pathway leads to aggravation of OGD-induced neuronal damage, and the expression of activin A and Smad3 is significantly downregulated (Xue et al., 2016). These results suggest that neuronal damage, induced by OGD, activates the activin A/Smad pathway, which exerts a neuroprotective role through the inhibition of apoptosis. Upregulation of RII may be the initiating factor in the activation of the activin A/Smad pathway induced by OGD injury, which may rely on an activin A positive feedback regulation mechanism (Table 2).

**Table 2:** Activin A targets in brain injury.

**activin A-related brain injury**	**Targets/mechanism**	**References**
Hypoxic-ischemic brain damage	Follistatin/BMP-4, Act A/Smad pathway, p-Smad3/CHOP/caspase-12, LC3II/Beclin1, JNK1/p38	Tian et al., 2014 Nakajima et al., 2014 Guo et al., 2014 Wang et al., 2016a Xue et al., 2017.
Focal cerebral ischemia	Act A/Smad pathway	Mukerji et al., 2009 Hiratochi et al., 2007 Xue et al., 2016.
Ischemic tolerance	Act RII/JNK1/Smad3/Smad4	Xue et al., 2016 Wang et al., 2016b.
Cerebral hemorrhage	activin A binding protein	Nicolas et al., 2017 Ebert et al., 2006.
Premature infant brain injury	Acvr2a/Acvr2b, IL-10	Dillenburg et al., 2018 González-Domínguez et al., 2016 Petrakou et al., 2013.
Sepsis encephalopathy	TNF-α/IL-6/IL-1, caspase-1/IL-1β/ IL-8	Tania et al., 2014 Petrakou et al., 2013 Asashima et al., 1991.

*Act A, activin A; Act RII, activin A receptor type II; Acvr2a/Acvr2b, activin A receptor type 2a/2b; BMP-4, bone morphogenetic protein 4; CHOP, C/EBP homologous protein; IL, interleukin; JNK1, c-Jun N-terminal kinase 1; LC3II, microtubule-associated protein light chain 3 II; TNF-α, tumor necrosis factor alpha*.

### Activin A signaling pathway and ischemic tolerance

Sublethal ischemic damage induces increased anti-ischemic response in the late stages of brain injury. This phenomenon is called ischemic tolerance (IT) and is induced by ischemic pre-conditioning (IPC) (Nishio et al., 2000). Ischemic brain injury can induce high expression of activin A, which can then activate an endogenous neuroprotective signaling pathway (Wu et al., 1999). It was demonstrated that after IPC in PC12 cells, the expression of activin A RII is upregulated, suggesting that the IPC-induced IT is mediated by the signaling pathway involving activin A, RII, and downstream Smad proteins (Xue et al., 2016). The main mechanism of IPC involves JNK1 activation, which inhibits Smad3 phosphorylation and the entry of the downstream Smad4 complex into the nucleus (Wang et al., 2016b). Furthermore, pre-treatment with JNK1 inhibitors also induces IT in PC12 cells. Given the existing crosstalk between the intracellular JNK1 protein and activin A/Smad pathway, JNK1 inhibitors may represent potential therapeutic agents for drug-induced IT (Wang et al., 2016b; Table 2).

### Activin A and cerebral hemorrhage

Studies have found that activin A is an immunosuppressive factor that induces cardiovascular and cerebrovascular diseases by inhibiting the activity of T lymphocytes in the body (Zipori and Barda-Saad, 2001; Ofstad et al., 2013; Yoon et al., 2013). Patients with cerebral hemorrhage show a significant increase in activin A and activin A binding protein levels, while patients with cerebral infarction show a significant increase in activin A (Ebert et al., 2006; Nicolas et al., 2017). Therefore, activin A and activin A binding protein levels in peripheral blood may be associated with cerebrovascular disease occurrence and development. Thus, detecting changes in these proteins may be helpful in guiding clinical diagnosis and treatment of cerebrovascular diseases, treatment decisions, and prognosis (Table 2).

### Activin A and premature infant brain injury

Brain injury in pre-term infants is mainly restricted to white matter damage. Its primary neuropathological feature is damage of oligodendrocyte precursor cells (OPCs), which inhibits the formation of myelin (Yue et al., 2018). A study found that the activin A receptor subtype Acvr2a/Acvr2b signaling pathway is involved in the regulation of myelin repair after brain injury in pre-term infants (Dillenburg et al., 2018). Acvr2b competes with Acvr2a for binding to activin A, thus resulting in decreased levels of Acvr2a-bound activin A, which in turn hinders Acvr2a-driven oligodendrocyte (OL) differentiation and myelination. Therefore, the activin A receptor Acvr2a may serve as a novel therapeutic target for the repair of myelin damage (Dillenburg et al., 2018).

activin A levels in the serum are significantly increased in pre-term infants during infection, which significantly inhibits the release of pro-inflammatory mediators from stimulated neonatal peripheral blood mononuclear cells *in vitro* and is associated with a dramatic increase in IL-10, an anti-inflammatory and immunoregulatory mediator (Petrakou et al., 2013; González-Domínguez et al., 2016). This suggests that activin A and IL-10 have strong anti-inflammatory and immunomodulatory effects in neonatal infection and are crucial for controlling the inflammatory response in neonates. Thus, activin A may be a target for the treatment of brain damage in prematurely born infants (Table 2).

### Activin A and sepsis encephalopathy

One of the pathogenic mechanisms of sepsis encephalopathy is the activation of inflammation and apoptosis, for which TNF-α and IL-6 are the two most important inflammatory cytokines, produced in the early stages of this disease (Sun et al., 2017). activin A promotes the expression of TNF-α, IL-6, and IL-1, in inflammatory and immune reactions, and eventually promotes the occurrence of inflammatory responses (Tania et al., 2014). In addition, studies have shown that serum activin A is elevated during acute and chronic inflammation, which may further increase the uninhibited inflammatory response leading to multiple organ failure and even death (Lee et al., 2016). However, other studies have indicated that activin A inhibits the inflammatory response by inhibiting caspase-1, IL-1β, and IL-8, thus leading to the dramatic increase in the production of the anti-inflammatory mediator IL-10 (Sierra-Filardi et al., 2011; Petrakou et al., 2013). Therefore, activin A has both pro-inflammatory and anti-inflammatory functions, is associated with the severity of sepsis encephalopathy, and can be used as an early predictor of this pathogenesis (Table 2).

## Treatment of brain injury targeted to activin A

### Exogenous activin A in the treatment of white matter damage

White matter damage is characterized by myelin injury, mainly affecting OLs (Liu X. B. et al., 2013). One study found that activin A, as a neurotrophic factor, plays a role in the repair of white matter damage (Dutta et al., 2014). Moreover, in an *in vitro* study, human recombinant activin A was added to cultures of primary OLs (no axons) or to neuro-glia co-cultures (Goebbels et al., 2017). After 3–5 days in culture, the number of OPCs in primary OL cultures that differentiated into mature OLs was significantly higher in treated versus untreated cells. Moreover, in the neuron-OL co-culture, the area of myelin was significantly higher in the activin A-treated cells. Thus, activin A seems to act as a myelination promoting factor. Researchers also found that activin A promotes the differentiation of OPCs into OLs by activating the ERK1/2 MAPK signaling pathway (Goebbels et al., 2017).

### Exogenous activin A in hypoxic-ischemic brain injury treatment

Using the hypoxia-ischemia brain damage model in neonatal rats, it was shown that pathological changes in the brain are significantly reduced by an intraperitoneal injection of activin A, confirming that the application of activin A reduces brain tissue damage induced by hypoxia-ischemia (An et al., 2005). In addition, in the OGD/reperfusion model in primary neuronal cultures, researchers used neuroprotector stress-induced phosphoprotein 1 (STI1) to treat neurons and found that STI1 is dependent on activin-A receptor 1 (ALK2) for the inhibition of neuronal apoptosis (Beraldo et al., 2018). Thus, since ALK2 acts as a downstream mediator of the STI1 neuroprotection pathway, it may be useful as a therapeutic target for ischemic brain injury (Beraldo et al., 2018). It was also shown that activin A reduces brain edema and the release of inflammatory cytokines in neonatal rats after hypoxic-ischemic brain damage and plays a role in nerve regeneration and functional repair, by increasing nestin protein expression, as well as the number and differentiation of neural stem cells (Peng et al., 2006; Zhang et al., 2016).

### Exogenous activin A in the treatment of focal cerebral ischemia-reperfusion injury

Focal cerebral ischemia and reperfusion lead to complex interactions between cells and molecules that eventually cause cells to either repair themselves or be destroyed (Cole et al., 1992; Li et al., 2004). Studies have found that activin A is an early response gene for cerebral ischemia and supports the survival of cortical neurons *in vitro* (Mukerji et al., 2009). In addition, activin A treatment protects neurons in the ischemic cerebral hemisphere, decreases the number of activated microglia, and reduces the activation of terminal kinases, like p38 and c-Jun N, involved in neuronal apoptosis after stroke. These findings suggest that activin A promotes tissue survival after focal cerebral ischemia/reperfusion.

### Emodin neuroprotection-targeting activin A pathway

One study demonstrated that administration of emodin in OGD-treated neuronal cultures significantly increases cell viability and activin A content in the culture medium, whereas it significantly decreases the expression of caspase-3 (Guo et al., 2013). Therefore, emodin promotes the up-regulation of activin A expression, increases the viability of neurons, and inhibits neuronal apoptosis in ischemic and hypoxic conditions, thus playing a neuroprotective role.

## Conclusion

In summary, activin A, as a neuroprotective factor, maintains the survival of neurons in the central nervous system, protects neurons from neurotoxicity, and plays important roles in brain injury (Iwahori et al., 1997; Keelan et al., 2000). Therefore, it can be used as a clinical test index, which is of great significance for disease diagnosis and prognosis (Bergestuen et al., 2010). The protective effect of the activin A-mediated signaling pathway against brain injury and its application in the clinic have received increased attention. Future research on the molecular mechanisms involved in activin A neuroprotection will provide new insight for developing treatments against brain damage.

## Author contributions

XS is the primary author who contributed to the conception and design of the review and the drafting of this article. LH and DX critically reviewed the article for some intellectual content. YQ and DM contributed to approving the final version of the manuscript submitted for publication.

### Conflict of interest statement

The authors declare that the research was conducted in the absence of any commercial or financial relationships that could be construed as a potential conflict of interest.
